# Baihu Jia Renshen Decoction Improves Type 1 Diabetic Rats by Modulating Metabolic Profile

**DOI:** 10.1155/ije/2139427

**Published:** 2025-08-18

**Authors:** Shufang Chu, Deliang Liu, Hengxia Zhao, Ling Liu, Juntong Li, Gaoxiang Wang, Xuemei Liu, Huilin Li

**Affiliations:** ^1^Department of Endocrinology, Shenzhen Traditional Chinese Medicine Hospital, Shenzhen 518033, China; ^2^Department of Endocrinology, Shenzhen Traditional Chinese Medicine Hospital Affiliated to Nanjing University of Chinese Medicine, Shenzhen 518033, China; ^3^Department of Endocrinology, Jiangsu Province Hospital of Chinese Medicine, Affiliated Hospital of Nanjing University of Chinese Medicine, Nanjing 210000, China

**Keywords:** Baihu Jia Renshen Decoction, metabolomics analysis, pancreatic tissues, traditional Chinese medicine, Type 1 diabetes mellitus

## Abstract

**Background:** Baihu Jia Renshen Decoction (BJRD) applied to diabetes mellitus (DM). We explored the metabolic regulatory pathways and mechanisms of BJRD in Type 1 DM (T1DM).

**Methods:** T1DM rat model was established with STZ induction and then continuously treated with insulin and different concentrations of BJRD for 2 weeks. The therapeutic effect of BJRD was evaluated by detecting the levels of fasting blood glucose and insulin in the blood and observing the ultrastructure of pancreatic tissues by transmission electron microscopy and immunofluorescent staining. Metabolomics analysis on rat serum was performed.

**Results:** Compared with the diabetes model (DC) group, the high-concentration BJRD treatment (BJRD-H) group reduced fasting blood glucose, increased insulin levels, and increased the quantity of β-cells in the pancreatic tissues in rats. And rat pancreatic islet β-cells in the DC group had lost their nuclear membranes, had abnormal mitochondrial morphology, and had significantly reduced secretory granules, whereas rat pancreatic islet β-cells in the BJRD-H group had moderately increased secretory granules, and the structure of the nucleus was apparently normal. Moreover, the metabolic profile expression pattern of rats in the BJRD-H group was closer to that of normal rats, suggesting that a high concentration of BJRD combined with insulin treatment was more effective. And differentially expressed metabolites were predominantly enriched in amino acid metabolism, including amino acids, glycine, serine, and threonine metabolism.

**Conclusion:** BJRD facilitated the therapeutic effect of insulin in T1DM rats and resulted in a significant improvement in their metabolic profiles, expanding the application of traditional Chinese medicine's alternative therapies in T1DM.

## 1. Introduction

Diabetes mellitus (DM) is characterized by glucose metabolism disorders resulting from various pathologies [1, 2]. Type 1 DM (in Type 1 DM [T1DM]) represents a significant subtype within the broader classification of DM caused by a lack of endogenous insulin secretion from pancreatic β-cells [3]. The β-cells have the ability to detect glucose and secrete insulin to regulate blood glucose within a relatively limited scope [4]. Therefore, upon destruction of these cells, patients with T1DM lose control of their blood glucose, and their blood glucose levels rise, causing harmful disorders of carbohydrate and lipid metabolism, which can lead to acute conditions and secondary complications that can jeopardize human health [5, 6].

T1DM usually develops in children or young adults, and the peak age of diagnosis is becoming younger as the incidence increases [7]. It has been shown that there is a significant reduction of β-cells in the pancreas in T1DM, as well as a large infiltration of inflammatory cells [8, 9]. Continuous exogenous insulin therapy stands as the prevailing treatment modality for individuals afflicted with T1DM, but exogenous insulin does not mimic normal endogenous insulin secretion in the body and may not even control the development of diabetes and its consequences [10, 11]. Exogenous insulin tends to cause hypoglycaemia, which in turn damages brain cells and leads to symptoms of dementia, seriously endangering the patient's life and health [12, 13]. Therefore, there is a need to find a therapeutic regimen that enhances the efficacy of exogenous insulin with few side effects through the combination of multiple drugs.

Chinese medicine has gained growing recognition in recent years for its role in preventing and treating chronic conditions like DM. To date, about 90 proprietary Chinese medicines for DM treatment have been certified by the National Institute of Medicinal Products (NIMP) [14]. Fufang-Zhenzhu-Tiaozhi capsule (FTZ) is a clinical, commonly used drugs preparation of Chinese medicine, have fall blood sugar, blood lipid, anticoagulant, and anti-inflammatory effect, for the treatment of T1DM [15]. Baihu Jia Renshen Decoction (BJRD) is from “Shanghan Lun”, which consists of g*ypsum*, *anemarrhena*, *licorice* and *ginseng*, and can be used to treat diseases with fever, sweating, and thirst as the main symptoms [16]. In accordance with the underlying pathogenesis of DM, BJRD can be used to treat it, especially as a complementary therapy. Studies have demonstrated that *anemarrhena* exhibits the potential to augment glucose consumption and glycogen synthesis in HepG2 cells that are resistant to insulin, thereby demonstrating hypoglycemic properties [17]. *Ginseng* reduces leptin and neuropeptide Y concentrations and appetite, lowers blood glucose, and improves insulin resistance [18]. *Licorice* has the effects of tonifying the spleen, benefiting the qi, clearing heat, removing toxins, and improving respiratory function [14]. Therefore, BJRD has been extensively utilized in the clinical treatment of diabetes with remarkable effectiveness. However, the multicomponent and multitarget properties of BJRD play a common role in efficacy, making the intrinsic mechanism of BJRD difficult to clarify.

In the field of Chinese medicine research, the evaluation of Chinese medicine efficacy is an important prerequisite for the search for active substances and quality markers in Chinese medicine, and metabolomics can provide important insights for the discovery of diagnostic markers, toxicity assessment, and disease pathogenesis [19, 20]. Metabolomics has also been used to explore the mechanisms of Chinese medicine in DM [21]. Urinary metabolomics investigations have revealed that Huanglian decoction modulates glyoxylate and dicarboxylic acid metabolism through upregulation of glucose transporter 4 (GLUT4), insulin receptor (INSR), and mitogen-activated protein kinase 1 (MAPK1) levels, thereby exhibiting therapeutic effects against T2DM [22]. In addition, metabolomic analysis confirmed that the combined extract of Scutellariae Radix and Coptidis Rhizoma had a significant ameliorative effect on T2DM by modulating the activities of pro-inflammatory cytokines, the MAPK pathway, the insulin signaling pathway, and enzymes related to glucose metabolism [23]. There is no metabolomics study on BJRD for the treatment of T1DM. Therefore, conducting metabolomics studies on BJRD for T1DM will be of great benefit in revealing the efficacy and mechanism of action of this traditional Chinese medicine.

In this study, rats were treated with varying concentrations of BJRD, and high-performance liquid chromatography-mass spectrometry (HPLC-MS) was used to analyze blood metabolomics. It is expected that the mechanism of action of BJRD for T1DM will be explored and revealed.

## 2. Materials and Methods

### 2.1. Preparation of BJRD

5500 g of gypsum (s1417814), 1780 g of anemarrhena (z2319411), 1100 g of ginseng (r0719116), 660 g of licorice (g0419515), and 990 g of japonica Rice were decocted with water twice for 30 min each, concentrated and cooled, and then prefrozen in the refrigerator at −80°C overnight. The decoction was dried through a vacuum freeze-dryer (the crude amount of lyophilized powder was 9.046 g decoction/g lyophilized powder). The drugs were purchased from the Chinese Medicine Decoction Factory of the Guangdong Provincial Pharmaceutical Materials Company. Japonica rice was selected as northeastern rice from COFCO Fulinmen.

### 2.2. Animals Treatment and Blood Glucose Testing

Male standard deviation (SD) rats of 7–8 weeks of age and weighing 160–200 g, were acclimatized and fed for 1 week at room temperature with a regular diet and water. The rats were randomly allocated into five groups, with six rats assigned to each group. T1DM model rats were induced by a subcutaneous injection of streptozotocin (STZ, 60 mg/kg), which was dissolved in sterile citrate buffer. The diabetes model (DC) group was then injected with sterile citrate buffer. Then insulin and BJRD were started after successful modeling, and the treatment lasted for a period of 2 weeks. After successful STZ induction, insulin injection (8 IU/kg/d) was given as the INS group; insulin injection and a low concentration of BJRD (8.2 g/kg/d) were given as the BJRD-L group; and insulin injection and a high concentration of BJRD (16.4 g/kg/d) were given as the BJRD-H group. At the end of the drug treatment, all rats were fasted for 12 h. Blood specimens were measured for blood glucose levels using glucometers (Roche, Switzerland) and performed according to the IACUC handbook (third edition).

### 2.3. Insulin Detection

Fasting insulin levels were detected in rats using an ELISA kit (CSB-E05070r, Shunyuan Biological, Shanghai). Serum was obtained by collecting it in serum separator tubes and allowing it to clot at room temperature for a duration of 2 h and centrifuging. Then the serum was withdrawn and diluted 200 times. Each well was incubated with 100 μL of standard and serum samples at 37°C for 1 h. The liquid in the wells was discarded, 200 μL of wash buffer was added and stood for 2 min, and then the liquid was discarded. Wash three times. Following the final rinse, the plate was turned over and tapped on a clean paper towel to remove as much liquid as possible. 100 μL of biotin antibody was added. Wash three times, as above. Inject 90 μL of TMB substrate and allow it to incubate for a duration of 30 min while maintaining darkness. Add 50 μL of termination solution and mix by tapping gently. Absorbance values were measured at 450 nm. To determine the insulin concentration in serum samples, the standard curve was generated and then fitted using Curve Expert software.

### 2.4. Immunofluorescence Experiment

Three rats from each group were selected at random, and the tail of their pancreas was chosen. The selected pancreas tails were then fixed in a 4% paraformaldehyde solution, followed by embedding and sectioning into 5-mm-thick paraffin sections. The paraffin sections of the pancreas were deparaffinized to water by xylene and gradient alcohol. The sections were immersed in a sodium citrate buffer solution with a concentration of 0.01 M and boiled for antigen repair using the autoclave; after stopping the heating, they were naturally cooled to room temperature. 0.3% Triton-X100 was added dropwise to the tissue sections for 10 min. After three washes with PBS, the sections were sealed with a 5% BSA solution. Then the blocking solution was discarded and primary antibodies were added: antirabbit glucagon (CST 2760) and antimouse insulin (CST 8138), and the sections were incubated overnight at 4°C. The sections were washed with PBS three times, and the corresponding fluorescently labeled secondary antibodies, Alexa Fluor 594 Goat antimouse (H + L) (A11012, Invitrogen) and IF488-Tyramide (tsa) (G1231, Xavier), were added for 1 h. Following three washes with PBS, the sections were blocked by adding glycerol containing DAPI, placed under the OLYMPUS FV3000 confocal fluorescence microscope, and photographed.

### 2.5. Transmission Electron Microscopy (TEM)

The procedure of electron microscopy was carried out in accordance with the previous description [24]. Three rats from each group were chosen at random and their pancreatic tails were selected. The tails were then fixed using 2.5% glutaraldehyde solution and embedded using the propylene oxide system. Afterward, ultrathin sections measuring 50 nm were cut on Leica UC-7 ultramicrotome. After staining with uranyl acetate (22,400, EMS) and lead citrate (19,314, TED PELLA), the sections were examined for the ultrastructure of rat β-cells using a TEM (JEM-1400 PLUS).

### 2.6. Metabolome Analysis

100 μL of blood sample from each group of rats was added to 400 μL of precooled methanol, mixed with shaking, and centrifuged. The liquid portion of the specimens was gathered, concentrated, and dehydrated using a vacuum. To obtain the samples for testing, 150 μL of 2-chlorophenylalanine (4 ppm) in 80% methanol was added and the mixture was filtered through a 0.22 μm membrane. Next, a calibration was performed by mixing 20 μL of each sample to be tested with a quality control (QC) sample. Subsequently, all samples were then subjected to LC-MS. The LC instrument was a Thermo Ultimate 3000 with a flow rate of 0.25 mL/min and a column temperature of 40°C. 2 μL sample was injected for gradient elution, and the mobile phases were 0.1% formic acid in water-0.1% formic acid in acetonitrile for the positive ion and 5 mM ammonium formate in water-acetonitrile for the negative ion. The Thermo Q Exactive MS had a positive ion spray voltage of 3.50 kV and a negative ion spray voltage of 2.50 kV. It also utilized a sheath gas of 30 arb and an auxiliary gas of 10 arb.

The raw data were converted to xcms input file format by Proteowizard software (v3.0.8789) and imported into R (v3.3.2) to obtain mass-to-charge ratio (m/z) and retention time (rt) and peak area (intensity) information. The chromatographically separated components continuously enter the mass spectrometry, and the base peak chromatogram (BPC) is obtained using the ion intensity as the vertical coordinate and time as the horizontal coordinate. When performing MS-based metabolomics studies, QC samples were used for QC during the assay, including the distribution of QC samples and relative SD (RSD) on the PCA analysis plot. Data were processed for self-adaptive (UV) permutation using orthogonal partial least squares discriminant analysis (OPLS-DA). Differential metabolite screening conditions were *p* value ≤ 0.05 and VIP ≥ 1. Accurate metabolite information was obtained by using the Human Metabolome Database (HMDB) (http://www.hmdb.ca) and further matches in Panomic's self-constructed standard database. Hierarchical clustering was done on the differential metabolites using cohesive hierarchical clustering, and the results were normalised and represented as heat maps. The metabolic pathways of the differential metabolites were also analysed by the Kyoto Encyclopedia of Genes and Genomes (KEGG).

### 2.7. Statistical Analysis

All data were expressed as mean ± SD. To distinguish significant differences relative to the NC group, one-way analysis of variance (ANOVA) followed by Duncan test was performed using SPSS 22.0 software. The level of significance was *p* < 0.05.

## 3. Results

### 3.1. BJRD Promotes the Therapeutic Effect of Insulin in T1DM Rats

**Figure 1:**
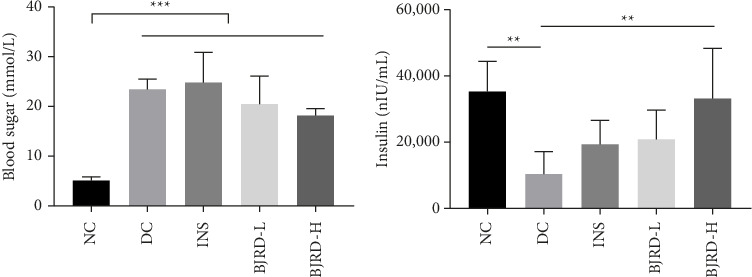
The changes of fasting blood glucose and insulin in serum. The changes of fasting blood glucose (a), insulin (b) levels in rats with different treatments. ^∗∗^*p* < 0.01, ^∗∗∗^*p* < 0.001.

All four groups of rats, except the normal group, were treated with drugs for 2 weeks after induction by STZ injection. We examined the content of fasting blood glucose and showed that compared with NC group, fasting blood glucose was significantly increased in DC, INS, BJRD-L and BJRD-H groups; compared with the DC group, insulin treatment slightly reduced the level of fasting blood glucose; compared with DC group, BJRD dose-dependently (combined with insulin treatment) reduced the elevated level of fasting blood glucose caused by injections of STZ, although there was no significant difference (Figure 1(a)). Compared with the NC group, fasting insulin levels in the DC group were significantly reduced; when compared to the DC group, fasting insulin levels were slightly increased in the insulin-treated group, whereas rats in the BJRD-H group had significantly higher fasting insulin levels (Figure 1(b)). These results suggested that high concentrations of BJRD contributed to the therapeutic effects of insulin in T1DM.

### 3.2. Effect of BJRD Combined With Insulin Treatment on β-Cells in T1DM Rats

**Figure 2:**
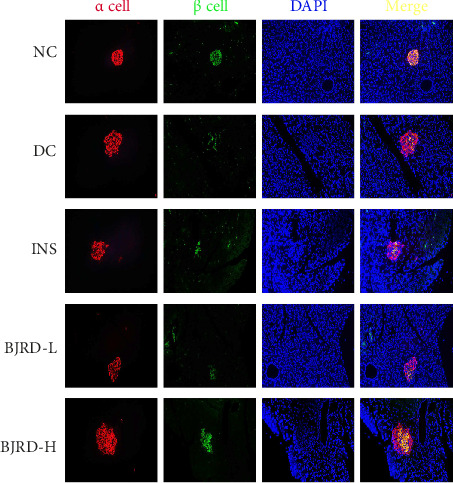
Expression of α-cells and β-cells in rat pancreatic tissues detected by immunofluorescence staining. 200x.

**Figure 3:**
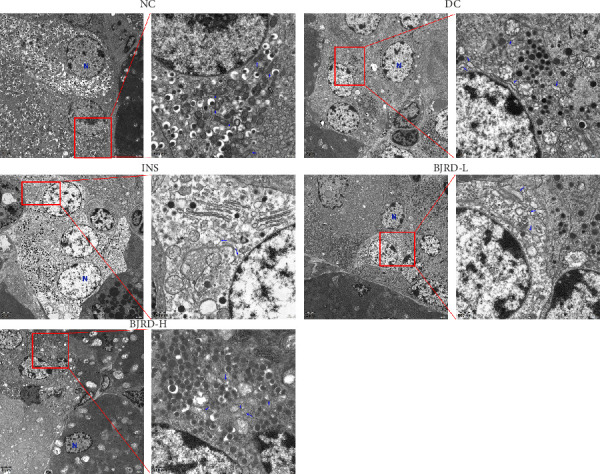
Ultrastructure of rat pancreatic tissue by transmission electron microscopy. N represents the nucleus, and the blue arrow represents the mitochondria.

To examine the impact of BJRD therapy on pancreatic β-cells in rats with T1DM, we detected both β-cell and α-cell marker proteins by immunofluorescence experiments. In contrast to the NC group, the DC group exhibited a substantial reduction in the quantity of β-cells. Insulin injection had no effect on rescuing β-cells, and a high concentration of BJRD combined with insulin rescued the number of β-cells (Figure 2). We also took pancreatic tissues from each group of rats for TEM to observe the β-cell ultrastructure. In the NC group, rats exhibited no pathological alterations in the pancreatic islet β-cells. The β-cells displayed normal characteristics in terms of nuclei, nuclear membranes, mitochondria, endoplasmic reticulum, and Golgi complex. Additionally, the cytoplasm contained numerous secretory granules that were evenly dispersed. In the DC group, pancreatic islet β-cells showed loss of nuclear membrane, vacuolization of mitochondria, and significantly fewer β-cell secretory granules than the NC group. While the overall number of secreted particles was elevated in the different treatment groups compared to the DC group, they were lower than in the NC group (Figure 3).

### 3.3. Metabolomics of BJRD Combined With Insulin Treatment on Serum of T1DM Rats

**Figure 4:**
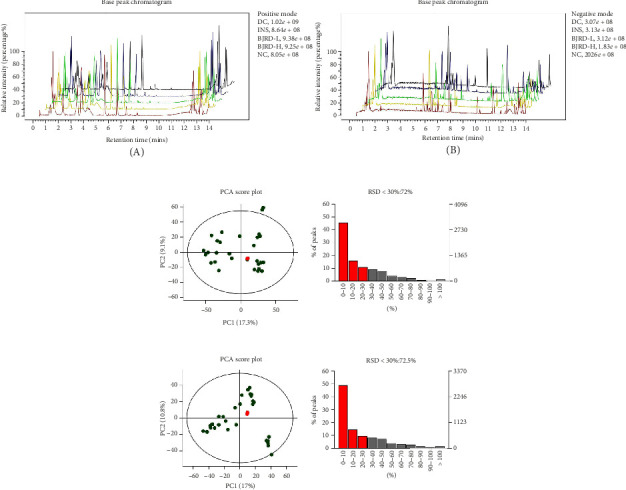
Quality control (QC) sample quality control analysis. (a) Base peak chromatograms (BPC) of samples in positive (A) and negative (B) ion modes for QC samples. (b–c) PCA scores and relative standard deviation (RSD) of QC samples in positive (b) and negative (c) ion mode.

**Figure 5:**
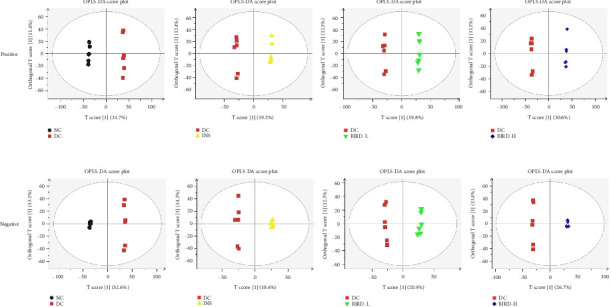
Orthogonal partial least squares discriminant analysis (OPLS-DA) score plots of NC group versus DC group, DC group versus INS group, DC group versus BJRD-L, DC group versus BJRD-H in positive (a) and negative (b) ion modes. NC, normal control group; DC, model group; INS, insulin treatment group; BJRD-J, insulin and low concentration of Baihu Jia Renshen Decoction treated group. BJRD-H, insulin and high concentration of Baihu Jia Renshen Decoction treated group.

First, the BPC of all samples was observed.  Figure 4(a) shows the BPC of the QC samples in typical positive and negative ion modes. It could be seen that the signal of all samples was strong and the peak capacity was large. For both positive and negative ions, the proportion of characteristic peaks with RSD less than 30% can exceed 70% in high-quality samples (Figures 4(b), 4(c)). The QC results above indicated that the variability caused by instrument error was minimal, affirming the reliability of the data quality. In addition, the OPLS-DA model was established. A clear separation between the DC group and the other groups was observed in both the positive and negative ion patterns (Figure 5), indicating significant differences in the metabolic profiles between them.

### 3.4. Effect of BJRD Combined With Insulin Treatment on Serum Metabolite Levels in T1DM Rats

**Figure 6:**
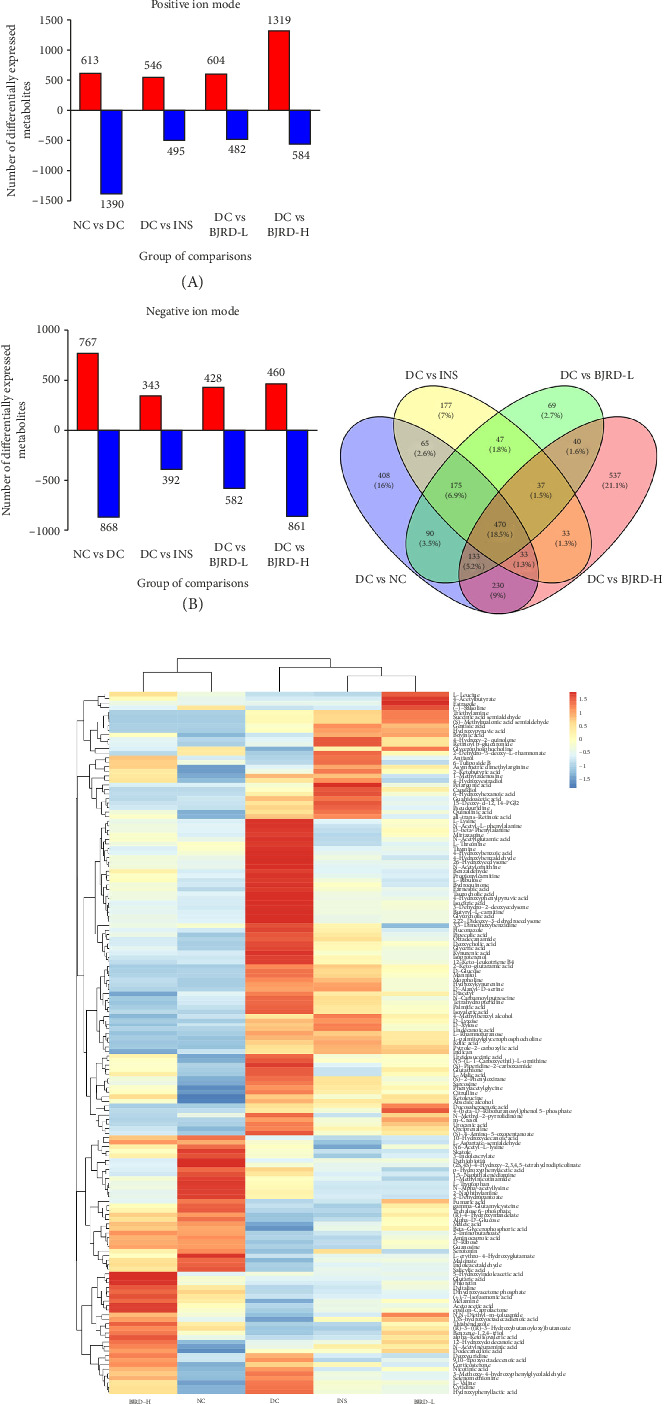
Analysis of differential metabolites in each group of rats. (a) Differences in metabolites in positive and negative ion modes for all groups. VIP value > 1 and the *p* value < 0.05. A: showed the metabolites in positive ion mode. B: showed the metabolites in negative ion mode. (b) Venn diagram of differential metabolites in each group of rats. (c) Hierarchical cluster analysis showing differences in metabolites in different groups. Each row represents metabolite and each column represents group.

The up- and downregulation of differential metabolites (VIP value > 1 and the *p* value < 0.05) in the absence of identification was further analyzed, and changes in differential metabolites in the positive ionic mode and in the anionic mode are shown in Figure 6(a). Subsequent identification of each differential metabolite revealed 248 differential metabolites in the NC-DC group, 134 differential metabolites in the DC-INS group, 140 differential metabolites in the DC-BJRD-L group, and 188 differential metabolites in the DC-BJRD-H group. Among them, 56 differential metabolites of the DC-INS group were the same as the NC-DC group, 90 differential metabolites of the DC-BJRD-L group were the same as the NC-DC group, and 107 differential metabolites of the DC-BJRD-H group were the same as the NC-DC group, suggesting that the metabolic pattern of BJRD-H would be more similar to that of the NC group (Figure 6(b)). Hierarchical clustering of metabolites at metabolic levels with relative values of metabolites under different experimental conditions to obtain heat maps (Figure 6(c)). It can be found that the expression pattern of the DC-BJRD-H group was closer to that of NC rats, indicating that a high concentration of BJRD combined with insulin treatment was more effective. In addition, we found that the differences in the expression of L-tryptophan and fumaric acid were more pronounced in the BJRD-H group compared with the DC group (Figure 6(c)), suggesting that the process of increasing insulin therapy for diabetes with high concentrations of BJRD may involve the L-tryptophan and fumaric acid metabolic pathways.

### 3.5. Metabolism Enrichment Analysis in DC and BJRD-H Groups

**Figure 7:**
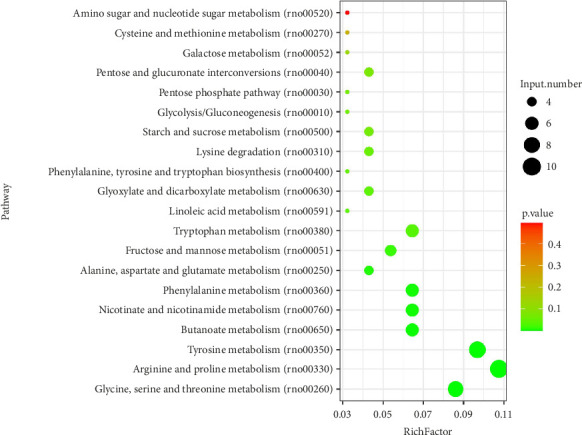
The bubble chart displays the metabolic pathways of the DC group and BJRD-H group as analyzed using KEGG enrichment analysis. DC, model group; BJRD-H, insulin and high concentration of Baihu Jia Renshen Decoction treated group.

According to hierarchical clustering analysis, the expression pattern of the DC-BJRD-H group was closest to that of the NC group. Therefore, we further performed KEGG analysis of the differential metabolites in DC-BJRD-H to search for potential metabolic pathways for the treatment of T1DM. The results revealed that these differentially expressed metabolites are predominantly enriched in several KEGG pathways associated with amino acids, including glycine, serine and threonine metabolism; arginine and proline metabolism; tyrosine metabolism; amino sugar and nucleotide sugar metabolism; and cysteine and methionine metabolism (Figure 7).  Table 1 shows the detailed information of Figure 7, in which, L-tryptophan participated in tryptophan metabolism, phenylalanine, tyrosine and tryptophan biosynthesis; fumaric acid participated in arginine and proline metabolism, tyrosine metabolism, butanoate metabolism, nicotinate and nicotinamide metabolism, phenylalanine metabolism, alanine, aspartate and glutamate metabolism.

## 4. Discussion

The prevalence of DM is anticipated to rise to 700 million individuals by 2045, making it one of the most challenging public health problems [25]. T1DM is a major subtype of DM, which is characterized as a metabolic disease characterized by insulin deficiency and disturbances in glucose metabolism caused by damage to pancreatic islet β-cells with vascular and neurological complications [26, 27]. A dearth of efficacious treatment options is evident, with the exception of lifestyle interventions and pharmaceutical interventions like insulin injection. Over the past few years, traditional Chinese medicine has shown a precise and distinct clinical impact in managing DM [28]. This study assessed the effectiveness of BJRD in combination with insulin for treating T1DM rats.

Insulin is essential for treating both T1DM and T2DM [29]. In this study, a T1DM rat model was constructed by intraperitoneal injection of STZ. Then, insulin was injected to treat T1DM for 2 weeks. However, due to the short duration of insulin treatment, as well as the small number of examples of mice in each group, compared to the DC group, the mice in the insulin-treated group had a decrease in blood glucose levels, an increase in insulin levels, and an increase in the number of pancreatic islet β-cells, but it was not significant.

BJRD is one of the earliest Chinese medicinal preparations used in the treatment of DM, and its ingredients include g*ypsum*, *anemarrhena*, *licorice*, *astragalus*, and *ginseng* [16]. Chinese medicine *ginseng* and *astragalus* granules may improve T1DM in autoimmune NOD mice by increasing insulin levels, lowering blood glucose levels, and attenuating immune cell infiltration [30]. Animal experiments have confirmed that BJRD can significantly reduce blood glucose in T2DM mice [16]. In this study, we constructed a T1DM rat model and tested different concentrations of BJRD in combination with insulin. Compared with low concentrations of BJRD, high concentrations of BJRD promoted the hypoglycemic effects of exogenous insulin, as evidenced by a decrease in fasting blood glucose, an increase in insulin levels, and an increase in pancreatic islet β-cell number and function in rats. This suggests that herbal BJRD combined with insulin has a more pronounced efficacy in the treatment of diabetes.

The etiology of T1DM is complex and its pathogenesis has not been fully elucidated. This study employed serum metabolomics and scientific data analysis to explore the potential mechanisms of BJRD in treating T1DM. By performing hierarchical clustering analysis of the differential metabolites, it could be visualized that the metabolic expression pattern of the BJRD-H group was closest to that of normal rats, which suggested that the diabetic rats recovered most strongly under the treatment of high concentrations of BJRD combined with exogenous insulin. Among these differential amino acids, L-tryptophan and fumaric acid were differentially expressed between the DC and BJRD-H groups. L-tryptophan is an essential amino acid that plays a crucial role in human health and disease and is involved in the biosynthesis of serotonin, melatonin and kynurenine, with the kynurenine pathway being associated with obesity and insulin resistance [31–33]. Studies have shown that L-tryptophan and kynurenine pathway metabolites are negatively associated with the incidence of T2DM [34]. Consumption of L-tryptophan-enriched chow from infancy in genetically diabetic rats maintained insulin secretion and delayed the development of T2DM [35]. Fumaric acid is a group of structurally simple compounds that exert antioxidant effects by inducing the transcription factor Nrf-2 [36]. Diabetic patients are prone to severe cardiovascular complications, diabetic cardiovascular disease is strongly associated with oxidative stress, and the antioxidant Nrf2 plays a key role in protecting the heart by reducing oxidative stress, making fumaric acid a potential drug for the prevention of diabetic cardiovascular complications [37]. Therefore, we speculated that the molecular mechanism of herbal BJRD combined with insulin for the treatment of T1DM maybe through L-tryptophan and fumaric acid, which deserves subsequent investigation.

KEGG enrichment analysis revealed that the differential metabolites were associated with many metabolic pathways, including glycine, serine and threonine metabolism; arginine and proline metabolism; tyrosine metabolism; amino sugar and nucleotide sugar metabolism; and cysteine and methionine metabolism. Amino sugars and nucleotide sugars are important carbohydrates in living organisms and participate in cell growth, differentiation, and metabolism as the building blocks of proteins and nucleic acids, respectively [38, 39]. For this reason, we speculate that BJRD combined with insulin treatment may restore the abnormalities of amino sugar and nucleotide sugar metabolism in T1DM rats, which also provides a new idea for us to explore the metabolic pathways affected by BJRD subsequently. In addition, reactive oxygen species (ROS) are particularly dangerous to sulfur-containing amino acids cysteine and methionine, which can disrupt protein outcomes and lead to cell death [40]. Studies have shown that high blood sugar promotes the overproduction of mitochondrial-derived ROS [41, 42]. The electron microscopy results in this study showed that mitochondria in pancreatic islet β-cells of T1DM rats were swollen and had abnormal morphology, whereas the mitochondrial morphology was normalized after treatment with a high concentration of BJRD in combination with insulin. This suggests that high-concentration BJRD combined with exogenous insulin treatment has the potential to restore pancreatic islet β-cell function by regulating cysteine and methionine metabolism, which deserves further investigation.

Therefore, we speculated that BJRD in combination with insulin plays an improvement role for T1DM, maybe through regulating the amino acids to control relative pathways. However, these thought are needed further study to confirm.

## 5. Conclusion

In summary, our study demonstrated that high concentrations of BJRD combined with insulin promoted the hypoglycemic efficacy of exogenous insulin and rescued the quantity and function of pancreatic islet β-cells. This is the first study to investigate the blood metabolomics of BJRD combined with insulin in the treatment of T1DM. This study found potential amino acids and metabolic pathways involved, which provide direction for our subsequent investigations.

## Figures and Tables

**Table 1:** The detail information of KEGG pathway in Figure 7 and batched amino acid ID.

Term	Matching IDs
Glycine, serine and threonine metabolism (rno00260)	C00078 C00109 C00168 C00188 C00213 C00258 C00441 C00581
Arginine and proline metabolism (rno00330)	C00122 C00213 C00327 C00436 C00437 C00441 C00581 C00624 C05942 C05947
Tyrosine metabolism (rno00350)	C00122 C00164 C00232 C00530 C00628 C00642 C01179 C03672 C05583
Butanoate metabolism (rno00650)	C00122 C00164 C00232 C00741 C01384 C04546
Nicotinate and nicotinamide metabolism (rno00760)	C00111 C00122 C00253 C01384 C02918 C03722
Phenylalanine metabolism (rno00360)	C00122 C00156 C00642 C00805 C03519 C05598
Alanine, aspartate and glutamate metabolism (rno00250)	C00122 C00232 C00438 C00940
Fructose and mannose metabolism (rno00051)	C00111 C00267 C00392 C02431 C03979
Tryptophan metabolism (rno00380)	C00078 C00637 C00780 C01717 C03722 C05635
Linoleic acid metabolism (rno00591)	C04056 C14762 C14825
Glyoxylate and dicarboxylate metabolism (rno00630)	C00149 C00168 C00258 C00311
Phenylalanine, tyrosine and tryptophan biosynthesis (rno00400)	C00078 C00441 C01179
Lysine degradation (rno00310)	C00047 C00408 C00489 C02727
Starch and sucrose metabolism (rno00500)	C00031 C00181 C00267 C00689
Glycolysis/Gluconeogenesis (rno00010)	C00031 C00111 C00267
Pentose phosphate pathway (rno00030)	C00031 C00121 C00258
Pentose and glucuronate interconversions (rno00040)	C00111 C00181 C00476 C00508
Galactose metabolism (rno00052)	C00031 C00111 C00267
Cysteine and methionine metabolism (rno00270)	C00051 C00109 C00441
Amino sugar and nucleotide sugar metabolism (rno00520)	C00031 C00181 C00267

## Data Availability

Data are included in the manuscript.
